# Cancer metastasis and EGFR signaling is suppressed by amiodarone-induced versican V2

**DOI:** 10.18632/oncotarget.5621

**Published:** 2015-10-21

**Authors:** Hung-Chieh Lee, Mai-Yan Su, Hao-Chan Lo, Chin-Chieh Wu, Jia-Rung Hu, Dao-Ming Lo, Tsu-Yi Chao, Huai-Jen Tsai, Ming-Shen Dai

**Affiliations:** ^1^ Institute of Biomedical Sciences, Mackay Medical College, New Taipei City, Taiwan; ^2^ Institute of Molecular and Cellular Biology, College of Life Science, National Taiwan University, Taipei, Taiwan; ^3^ Hematology/Oncology, Tri-Service General Hospital, National Defense Medical Centre, Taipei, Taiwan; ^4^ Hematology/Oncology, Shuang-Ho Hospital, Taipei Medical University, Taipei, Taiwan

**Keywords:** amiodarone, versican, metastasis, EGFR signaling

## Abstract

Extracellular matrix components play an active role in cancer progression and prognosis. Versican, a large extracellular matrix proteoglycan, can promote cancer metastasis through facilitating cell proliferation, adhesion, migration and angiogenesis. We had previously demonstrated that amiodarone caused ectopic overexpression of *similar to versican b* (*s-vcanb*), inhibited EGFR/GSK3β/Snail signaling, and enhanced Cdh5 at the heart field of zebrafish, indicating interference with epithelial-mesenchymal transition (EMT). Since S-vcanb is homologous to mammalian versican V2 isoform, we examined the effects of amiodarone on mammalian tumor proliferation, migration, invasion and metastasis *in vitro* and *in vivo* and on EMT signaling pathways. Monolayer wound assays and extracellular matrix transwell invasion assays showed reduced migration and invasion by 15 μM amiodarone treated B16OVA, JC, 4T-1, MDA-MB-231 and MCF-7 tumor cell lines. All cancer cell lines showed reduced metastatic capabilities *in vivo* after treatment with amiodarone in experimental animals. Western blots revealed that EMT-related transcription factors Snail and Twist were reduced and E-cadherin was enhanced in amiodarone treated cells through an EGFR/ERK/GSK3β-dependent pathway. Immunohistochemistry showed amiodarone lead to increased expression of versican V2 isoform concomitant with reduced versican V1. Our study illustrated the role of versican v2 in EMT modulation and cancer suppression by amiodarone treatment.

## INTRODUCTION

Cancer metastasis is the major cause of cancer-related mortality [1]. Discovering molecular targets that regulate metastasis or act as predicting/prognostic biomarkers is essential for developing effective therapies. Epithelial-Mesenchymal Transition (EMT) and Mesenchymal-Epithelial Transition (MET) have been thoroughly studied in mammalian development. Numerous embryonic events and developing organs depend on the switch between epithelial and mesenchymal phenotypes, such as gastrulation [2], neural crest formation [3] and heart valve formation [4]. EMT has been well implicated in cancer metastasis, characterized by loss of E-cadherin and increased expression of several transcriptional repressors of E-cadherin, such as Twist and Snail [5–8]. EMT in cancer stem cells has also been related to therapy resistance [9, 10]. Extracellular matrix components (ECM) also play an active role in tumor progression and are important determinants for the growth and progression of solid tumors [11]. Versican, a key ECM component, is a large chondroitin sulfate proteoglycan belonging to the lectican family [12–14]. Alternative splicing of versican generates at least four isoforms named V0, V1, V2, and V3. *In vitro* and *in vivo* studies reveal that Versican modulates cell adhesion, proliferation, and migration, and, hence, plays a central role in tissue development, as well as a number of pathologic processes [15–17]. Numerous studies have reported that the V1 isoform promotes cancer cell motility and invasion [18–20]. Both G1 and G3 globular domains in Versican V1 promote cell proliferation of NIH-3T3 fibroblasts and tumor cells [18, 19, 21, 22]. The Versican G1 domain is proposed to stimulate proliferation by destabilizing cell adhesion, while the G3 domain is proposed to induce proliferation by the interaction of its EGF-like motifs with EGFRs [23, 24]. Recently, Du *et al*. [25] found that overexpression of *versican* enhanced breast cancer self-renewal through EGFR/AKT/GSK3β signaling and conferred to chemotherapy resistance.

Amiodarone is a widely used antiarrhythmic drug which was approved by the US Food and Drug Administration in 1985 [26, 27]. It is considered a broad-spectrum antiarrhythmic agent [28] for the treatment of ventricular tachyarrhythmias and atrial fibrillation [29]. We previously reported that Amiodarone could inhibit zebrafish cardiac valve formation through inducing the expression *versican* and chromodomain-helicase-DNA-binding protein 5 tumor suppressor gene (*cdh5*; VE-cadherin) in the heart field. These data suggest that Amiodarone may have sufficient effect to interfere EMT processes during cardiogenesis [30]. In this study, we demonstrated that Amiodarone caused the ectopic expression of versican V2, conserved as zebrafish ortholog *s-vcanb*, and cancer growth/metastasis inhibition through EMT modulation and suppressing EGFR signaling.

## RESULTS

### Amiodarone inhibits migration and invasion of 4T-1 breast cancer cell line

**Figure 1:**
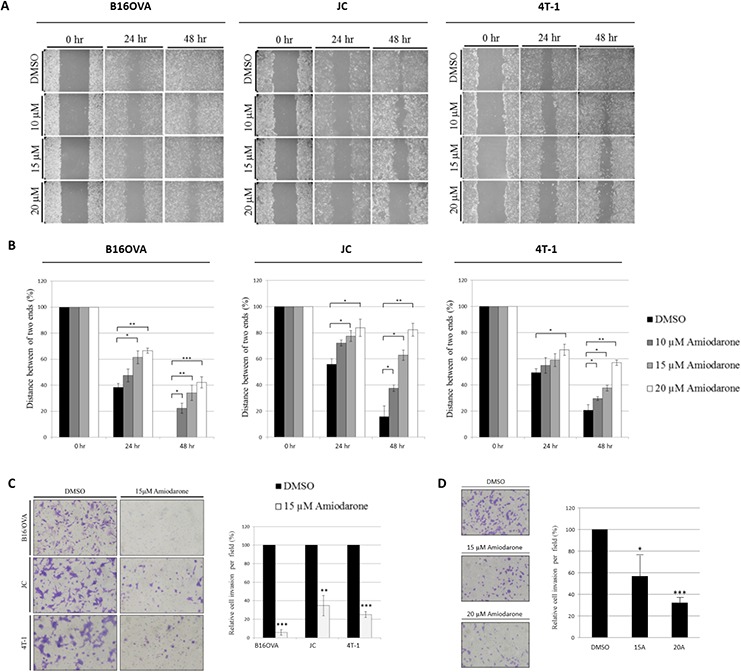
Amiodarone inhibits cancer cell migration and invasion **A.** Confluent monolayers of B16OVA, JC and 4T-1 cells were mechanically wounded with a pipette tip, and photos were obtained at 0 h, 24 h and 48 h after stimulation with 10, 15, or 20 μM of Amiodarone. DMSO treatment served as control. **B.** To quantify wound healing, cell numbers from three random fields within the wound were counted. **C.** Matrigel invasion assay of murine B16OVA, JC and 4T-1 cells treated with DMSO or 15 μM Amiodarone. To quantify matrigel invasion, five random fields were counted. **D.** Matrigel invasion assay of human breast MDA-MB-231 cells treated with DMSO, 15 μM or 20 μM Amiodarone. To quantify matrigel invasion, five random fields were counted. A representative experiment is shown in triplicate along with s.e.m. in B, C and D. Statistical significance was determined by Student's *t*-test. ***P < 0.001; **P < 0.005; *P < 0.01.

We first sought to establish that Amiodarone mediates a functional inhibition of cell migration and invasion using a monolayer wound healing assay. Quantification of cell movement over 24 and 48 hours showed that 15 μM Amiodarone inhibited migration of B16OVA by 34 ± 6%, JC by 63 ± 6%, and 4T-1 by 37 ± 2% (Figure 1A, 1B). Cell invasion was also examined using transwell migration assays. Amiodarone treatment significantly decreased the invasive capabilities in B16OVA, JC, 4T-1 (Figure 1C) and MDA-MB-231 cells (Figure 1D). The results of the transwell assay were consistent with those of the wound-healing assay, demonstrating that Amiodarone leads to suppression of the invasive capabilities of melanoma and breast cancer cells.

### Amiodarone induces ectopic versican V2 expression in cancer cells

**Figure 2:**
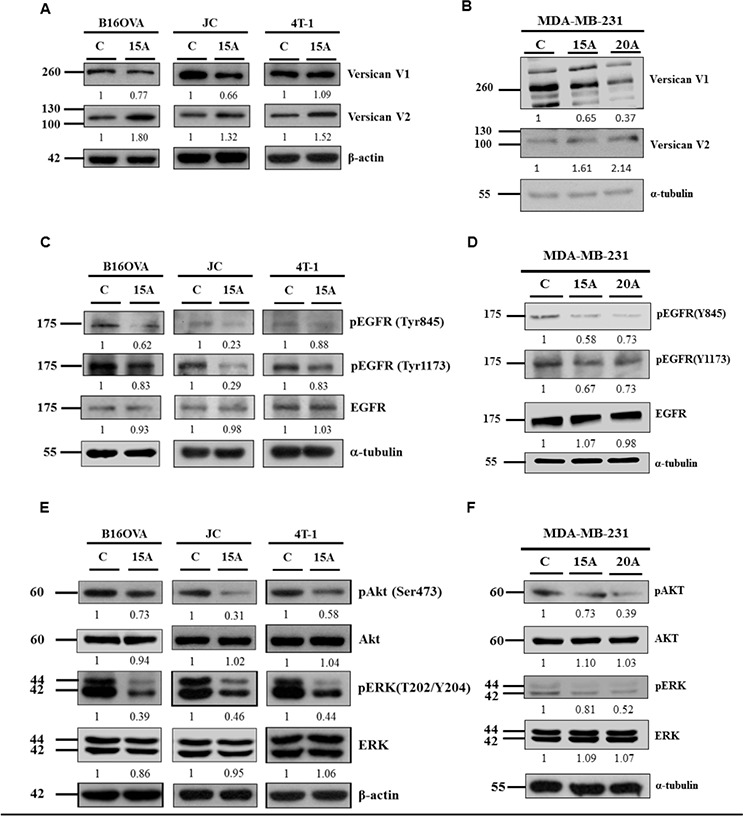
Amiodarone can induce Versican V2 expression, inhibit EGFR signaling and downstream AKT and ERK signaling Mouse tumor cells B16OVA, JC and 4T-1 cells **A, C, E.** and Human breast cancer cells MDA-MB-231 **B, D, F.** were treated with 15 uM or 20 μM Amiodarone (15A or 20A) for 24 hr and analyzed for Versican V1, V2 **A, B,** pEGFR (Tyr845), pEGFR (Tyr1173), EGFR **C, D.** pAKT (Ser473), AKT, pERK (T202/Y204) and ERK **E, F.** The relative intensities of each protein among control group (C) and Amiodarone-treated cells (15A) were as indicated. β-actin, and α-tubulin, were used as internal control.

In vertebrates, Versican isoform V1 increases EGFR signaling, whereas isoform V2 exhibits a biological activity opposite from that seen in V1 [33]. Since the observed functional similarity proposed that zebrafish S-Vcanb may be functionally conserved in mammalian versican V2, we examined the expression of Versican isoforms V1 and V2 in Amiodarone-treated cell lines B16OVA, JC, 4T-1 and MDA-MB231 cells. After 15 μM Amiodarone incubation for 24 hours, all treated cell lines showed increased V2 expression compared to control cells (Figure 2A, 2B). At the same time, V1 expression was reduced in the Amiodarone-treated B16OVA, JC and MDA-MB231 cells, but not in 4T-1 cells (Figure 2A, 2B). Amiodarone induced enhanced Versican V2 expression in 2 out 3 tested cancer cell lines.

### Amiodarone inhibits EGFR signaling in cancer cells

EGFR has been identified as an oncogene [34]. Anticancer therapies with mAbs such as cetuximab, that block the extracellular ligand binding domain prevent signaling molecules from attaching and activating the tyrosine kinase [35]. Therefore, we examined whether Amiodarone regulates the migratory and invasive phenotypes of cancer cells through EGFR pathways similar to that found in our previous work with zebrafish embryos (submitted to Cardiovascular Research). We analyzed the expression levels of phosphorylated EGFR, EMT markers and EMT-related factors by Western blot analysis. Firstly, we examined the effects of Amiodarone on the phosphorylation of tyrosine residues in EGFR. Phospho-Tyr-845 of EGFR is a known autophosphorylation site [36–40]. Phospho-Tyr-1173 serves as one of the docking sites for the cytosolic signaling molecule, Src homology 2 (SH2)-domain-containing transforming protein and growth factor receptor-bound protein 2 (GRB2) [41–43]. Treatment of B16OVA, JC, 4T1 and MDA-MB231 cells with Amiodarone for 24 hr resulted in decreased EGF-induced phosphorylation of EGFR on individual tyrosine residues (Figures 2C, 2D).

We also performed loss of function study to prove the pathway we demonstrated in this study. We evaluated the effect of inhibiting the kinase activity of EGFR with tyrphostin (AG1478; Supplementary Figure S2A). Results showed that the EGFR phosphorylations were increased significantly in the presence of EGF (50 ng/ml). On the contrary, the EGFR phosphorylations were significantly reduced in Amiodarone (15 μM) and AG1478 (30 μM) treated cells (Supplementary Figure S2A). Cetuximab is a chimeric (mouse/human) monoclonal antibody which binds to the extracellular domain of EGFR with high specificity. Therefore, we used Cetuximab, another EGFR blocking antibody, to perform loss of function study (Supplementary Figure S2B). Results showed that the EGFR phosphorylation were increased significantly when EGF (50 ng/ml) were added. However, the EGFR phosphorylation were reduced both in the cells treated in either or both the presence of Amiodarone (15 μM) and Cetuximab (75 μg /ml). The evidence presented indicates that Amiodarone can repress the phosphorylation of EGFR at multiple tyrosine residues, which should result in the inhibition of EGFR tyrosine kinase activity *in vitro*.

### Amiodarone represses EMT processes through both AKT and ERK signaling

EGFR signaling regulates cell motility through activation of EGFR's intrinsic kinases, leading to the activation of several downstream intracellular signaling pathways, including rat sarcoma–MAPK kinase (MEK), extracellular-related kinase (ERK), phosphoinositide 3-kinase (PI3K) and AKT pathways. For downstream signaling, our results showed that the total levels of AKT and ERK were unchanged in Amiodarone-treated B16OVA, JC, 4T-1 and MDA-MB231 cells (Figure 2E, 2F). However, phosphorylated ERK and AKT were reduced in the Amiodarone-treated cells, compared to non-treated control cells.

### Amiodarone induces E-cadherin via by inhibiting GSK3β

**Figure 3:**
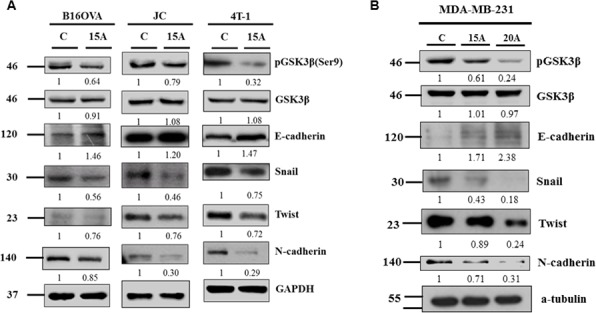
Evidence that amiodarone regulates EMT Mouse tumor cells B16OVA, JC and 4T-1 cells **A.** and Human breast cancer cells MDA-MB-231 **B.** were treated with 15 μM or 20 μM Amiodarone for 24 hr and analyzed for pGSK3β, GSK3β, E-cadherin, Snail, Twist and N-cadherin. The relative intensities of each protein among control group (C) and Amiodarone-treated cells (15A) were as indicated. β-actin, and α-tubulin, were used as internal control.

GSK3β signaling and E-cadherin expression is a hallmark of EMT transition, an important characteristic of metastatic potential. Amiodarone treatment indeed decreased GSK3β phosphorylation without changing the total GSK3β protein level (Figures 3A, 3B). Simultaneously the expression levels of epithelial marker E-cadherin increased whereas the mesenchymal markers N-cadherin, Snail and twist decreased in all tested cell lines (Figure 3A, 3B). Therefore, it is crucial to determine if the repression of these EMT markers by Amiodarone is dependent upon GSK3β activity. Accordingly, we incubated tumor cells with a GSK3β inhibitor, BIO, prior to treating with amiodarone and analysis of Snail and Twist. Supplementary Figure S3 shows that 1 μM BIO restored Snail and Twist and suppressed E-cadherin expression in amiodarone treated cells. Therefore, our results showed that Amiodarone repression of EMT marker expression, in particular Snail protein, is GSK3β-dependent.

### Amiodarone-induced Snail suppression is dependent on increased Versican V2 expression

**Figure 4:**
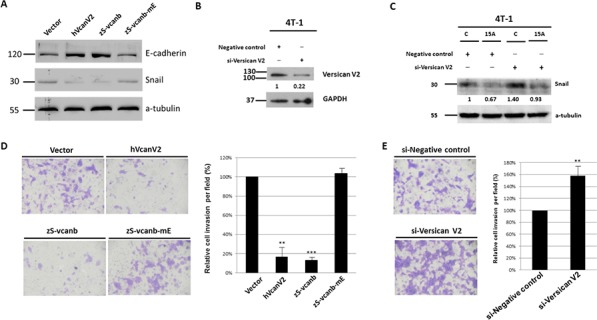
Versican V2 inhibits cancer cell invasion and metastasis **A.** Western blot analysis of E-cadherin and Snail in human Versican V2 (hVcanV2), zebrafish S-vcanb (zS-vcanb), and zebrafish S-vcanb-mE (zS-vcanb-mE) overexpressed cells. **B.** Western blot analysis of Versican V2 in Versican V2 knockdown cells. **C.** Western blot analysis of Snail in Versican V2 knockdown cells treated with DMSO (c) or 15 μM Amiodarone (15A). **D.** Matrigel cell invasion assay in human Versican V2 (hVcanV2), zebrafish S-vcanb (zS-vcanb), and zebrafish S-vcanb-mE (zS-vcanb-mE) overexpressed 4T-1 cells. **E.** Matrigel cell invasion assay in Versican V2 knockdown 4T-1 cells.

Gain-of-function and loss-of-function assays were performed to investigate the role Versican V2 plays in Amiodarone-mediated repression of EMT markers. Overexpression of versican V2 was achieved by transfection of 4T-1 cells with human VcanV2 or zebrafish Svcanb transgenes. Both of these transgenes each caused increased epithelial E-cadherin expression and decreased Snail protein expression (Figure 4A). In contrast transfection with a loss of function transgene, S-Vcanb-mE, failed to induce E-cadherin or reduce Snail expression (Figure 4B). We also demonstrate that the expressions of ERK, AKT and GSK3β, and Twist protein level were affected in the cell lines treated with recombinant si-Versican V2 and zS-vcanb-mE. Overexpression of versican V2 was achieved by transfection of 4T-1 cells with either human VcanV2 or zebrafish S-vcanb transgenes. All these transgenes resulted in the reduction of ERK and AKT activity due to the decreased levels of phosphorylated ERK (p-ERK) and pAKT. The protein level of Twist was reduced as well. In contrast, cells transfected with a loss-of-function of transgene, zS-vcanb-mE, were not able to affect the pERK, pAKT, and Twist protein levels. Thus, we concluded that Amiodarone-mediated ectopic expression of Versican V2 and activation of signalling pathways is also inhibited in recombinant si-Versican V2 and zS-vcanb-mE treated cells (Supplementary Figure S4).

Similarly, using siRNA to knockdown VcanV2 expression before amiodarone treatment, E-cadherin was reduced while Snail was increased (Figure 4C). We also demonstrated the loss of EGFR activity inhibition by Amiodarone upon knockdown VcanV2 (Supplementary Figure S5). The role of VcanV2 in this process was demonstrated by reduced invasion by cells harboring the transgene for human or zebrafish VcanV2 and increased invasion by cells harboring shRNA-VcanV2 (Figure 4D, 4E). Taken together, we concluded that the VcanV2-dependent effects of Amiodarone on EGFR signaling suppression

**Figure 5:**
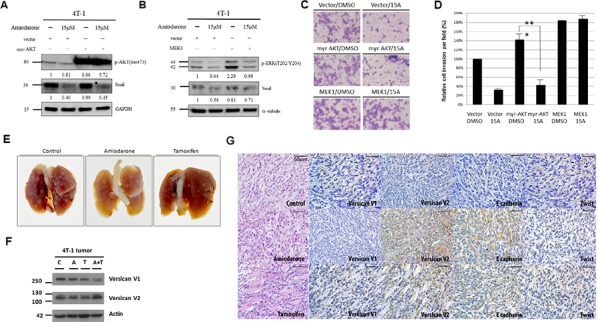
Amiodarone represses Snail protein expression through the ERK pathway and block metastatic properties *in vivo* Western blot analysis of AKT, ERK, and Snail phosphorylation in 4T-1 cells treated with DMSO(−) or Amiodarone (15 μM) and transiently transfected with **A.** myr-AKT- and **B.** MEK1-expressing vectors. GAPDH and α-tubulin were used as internal control. The relative intensities of each protein were as indicated. **C.** Matrigel invasion assay of 4T-1 cells transiently transfected with myr-AKT- and MEK1-expressing vectors and treated with DMSO or 15 μM of Amiodarone. **D.** To quantify matrigel invasion from C, five random fields were counted. A representative experiment is shown in triplicate along with s.e.m. Statistical significance was determined by Student's *t*-test. ***P < 0.001. **E.** balb/c mice were intravenously inoculated with 4T-1 cells via tail veins and then treated with DMSO, oral Amiodarone, or oral Tamoxifen. Mice were sacrificed after 21 days, and the whole lungs were excised for inspection of pulmonary metastasis. **F.** Tumor proteins were collected from the lung metastasis, and expression of Versican V1 and V2 was analyzed by Western blot. α-tubulin was used as internal control. **G.** Histological analyses of lung metastatic tumors by Versican V1, Versican V2, E-cadherin and Twist staining. Scale bars: 500 μm.

To confirm the role of ERK and AKT signaling pathways in Amiodarone-mediated EMT repression, we transfected vectors expressing constitutively activated MEK1 to activate ERK signaling and constitutively activated AKT (myr-AKT) into 4T-1 cells. We showed that the levels of pAKT and pERK were significantly increased in 4T-1/myr-AKT and 4T-1/MEK1 cells, respectively (Figure 5A, 5B). Amiodarone treatment still repressed Snail expression in 4T-1/myr-AKT cells (Figure 5A). In contrast, Snail level was restored in 4T-1/MEK1 cells (Figure 5A, 5B). Therefore, since the invasive capabilities of cancer cells were restored by MEK1, but not by myr-AKT (Figure 5C, 5D), it was concluded that ERK is involved in the regulation of Snail through a VcanV2/EGFR signaling axis.

### Amiodarone inhibits cancer metastasis *in vivo*

To determine whether this *in vitro* therapeutic effect can translate to experimental metastasis *in vivo*, we challenged balb/c mice with 4T-1 cells and then orally treated with PBS (control), tamoxifen (400 mg/kg/day) or Amiodarone (180 mg/kg/day). Mice were euthanized at day 21 and lungs were removed for analyses. The metastatic nodules in the excised lungs were significantly fewer and smaller in mice treated with either tamoxifen or amiodarone compared to the control animals. Metastatic extent was similar in tamoxifen and amiodarone treated groups (Figure 5E). Western blot analyses of VcanV1 and VcanV2 in tumor cells obtained from the diseased lungs confirmed that Amiodarone treatment induced VcanV2 and repressed V1 *in vivo* (Figure 5F). Immunohistocytochemical analyses of the excised lung from tumor-inoculated mice confirmed that Amiodarone treatment significantly enhanced VcanV2 and E-cadherin expressions, and suppressed VcanV1 and twist in tumor tissues (Figure 5G). Therefore, Amiodarone treatment is able to suppress breast cancer growth and metastasis *in vivo* by modulating Vcan isoform expression.

## DISCUSSION

**Figure 6:**
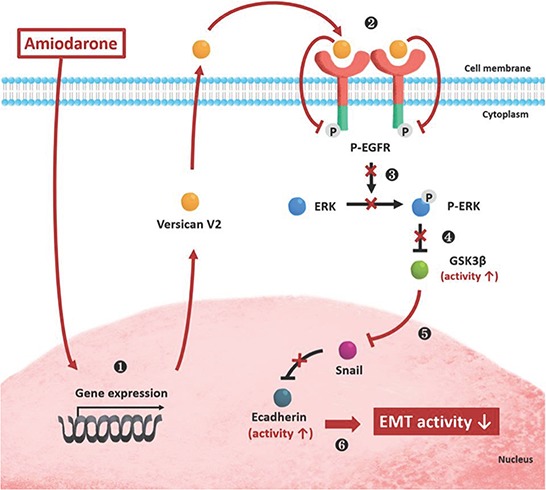
A schematic representation depicts effects of Amiodarone on cell signalling pathways, tumour invasion and metastasis formation **1.** Amiodarone induces ectopically VcanV2 transcription in nucleus, **2** resulting overexpressive production of VcanV2 protein in cytoplasm. **3.** VcanV2 interacts with EGFR receptor on ECM. **4.** VcanV2/EGFR interaction inhibits EGFR singalling through inhibiting the phosphorylation ERK. **5.** Inhibition of ERK activity induces the increase of GSK3β activity. **6.** Activation of GSK3β reduces the production of Snail protein. **7.** Finally, the protein level of E-cadherin is increased, resulting in reducing the EMT activity in cancer cells.

This is the first report to demonstrate that Amiodarone, a potassium channel blocker used as an anti-arrhythmic drug, can inhibit tumor cell proliferation and metastasis by inducing the VcanV2 isoform expression. This effect is accompanied by down-regulating EGFR signaling through ERK/GSK3β pathways. A consequence of this EMT transition blockade is up-regulation of E-cadherin. Overexpressed VcanV2 is the mediator, as it binds to and inhibits the action of EGFR through its EGF-like domains (Figure 6). This scenario and its participants are analogous to the amiodarone effects of EMT transition block we previously observed during heart valve development in zebrafish [30]. Moreover, we performed additional Matrigel invasion assay of dronedarone and amiodarone. Equivalent cancer cell invasion inhibition was shown in thess two compounds. Additionally, western blot analysis showed that dornedarone can induced VcanV2 ectopic expression in 4T-1 cells (Supplementary Figure S6). Therefore, iodine-free dronedarone may possibly be a safer alternative to amiodarone.

Many studies have proved that ECM components play an active role in tumor progression and are important determinants for the growth and progression of solid tumors [11]. In particular, increased expression of Versican, which has been reported in peritumoral stromal tissues, is correlated with histological tumor grade score, suggesting that this protein may be a strong factor in predicting disease relapse in lymph node-negative breast cancer patients [44]. Moreover, the role of Versican in modulating the loss of adhesion and cell motility has also been recognized in cases of breast cancer metastasis [22] or ovarian cancers [45].

Alternative splicing of Versican generates at least four isoforms, and these isoforms play distinct roles may due to differences in chondroitin sulfate (CS) domains [33]. V0 contains both CSα and CSβ; V1 and V2 possess only CSβ and CSα, respectively; and V3 contains neither CSα nor CSβ. The CSβ domain–containing V1 isoform can induces neuronal differentiation and promotes neurite Outgrowth [46]. Sheng et al., [33] also report that V1 isoform is implicated in cell proliferation enhancement, cell cycle progression and apoptosis resistance. In contrast, The CSα domain-containing V2 inhibits axon and neurite growth [47] and is associated with suppression of cell proliferation and causes inhibition of the EGFR signaling pathway [33].

Interestingly, Versican functions not only as a scaffold or substrate to be consumed during tumor cell invasion, but also represents a central component of cancer-related inflammation, as it can bind multiple types of cell adhesion receptors, growth factor receptors, and chemokines to provide a complex set of environmental cues to inflammatory and cancer cells in versican-rich sites [48]. For example, Ricciardelli et al., [21] demonstrated that prostate cancer cells *in vitro* could utilize the hyaluronan, and versican, secreted by prostatic fibroblasts to assemble a pericellular matrix and promote their motility. Versican treatment can induce ovarian cancer cell invasion through an ECM barrier, and suggest that formation of a CD44/hyaluronan/versican macromolecular complex promotes the motility and invasion of ovarian cancer cells [49, 50].

Therefore, manipulation of versican expression with relevant EGFR signaling alteration may provide novel therapeutic mechanism against EMT process in cancer (Figure 6). In this report, we found that Amiodarone can induce V2 isoform expression both *in vitro* and *in vivo*. The balance between VscanV1 and VcanV2 plays a role in modulation tumor biology. Although VcanV1 in JC cells was not reduced, EGFR signaling, which could be induced by VcanV1 in all the cells types we used, was reduced. Sheng *et al.* [33] proposed that VcanV1 exhibits a biological function opposed to that of VcanV2, whereas VcanV1 is able to enhance cell proliferation and VcanV2 inhibits cell proliferation instead, consistent with our finding. Thus, our data suggest a new approach for the modulation of Versican isoform synthesis for the development of therapeutics against cancer. Additionally, the opposing effects between VcanV1 and VcanV2, suggest that the expression amounts of these two isoforms may provide a suitable extracellular environment for normal proliferation and survival of cells. However, the intact regulatory mechanisms are still unclear. These findings prompted us to probe more deeply into the functions of Versican isoforms.

Accumulating evidence in recent years indicates that various processes associated with EMT occur during tumor progression and that these processes closely resemble those occurring in normal organ development. Several pathways involved in the developmental control of EMT are subverted during tumor formation by activation of oncogenic signaling and disruption of tumor suppressor networks [51]. Knowledge acquired in studying EMT processes may provide new clues to understanding the nature of invasion and metastatic cascades. Our previous work with zebrafish cardiac valve formation revealed that amiodarone upregulated versican, inhibited EMT transition, EGFR signaling and increased cadherin expression [30]. In this report, our data demonstrated that the effects of Amiodarone on the growth inhibition of tumor cells, recapitulates the effect on the same pathways as in zebrafish embryos. In summary, Amiodarone was shown to induce *VcanV2*, the mammalian ortholog gene of zebrafish *s-vcanb*, resulting in the inhibition of EGFR/ERK/GSK3β/Snail signaling and, consequently, the increase of E-cadherin, leading to the suppression of migration, invasion, and metastatic capabilities of breast cancer cells. Therefore, altered EGFR signaling is pivotal for cancer cell growth, invasion, and metastasis. Exploitation of mechanisms to upregulate endogenous VcanV2 may provide a novel anticancer treatment strategy through EGFR-PI3K/AKT signaling blockade.

## MATERIALS AND METHODS

### Cell culture

The murine cancer cell lines B16OVA (melanoma), JC, 4T-1(breast), and human breast cancer lines MDA-MB-231 and MCF-7 were obtained from the American Type Culture Collection (Rockville, MD, USA). Cells were cultured at 37°C in a humidified atmosphere of 5% CO2. All media were changed every 3 days.

### Plasmid construction

Polymerase chain reaction (PCR)-based *in vitro* mutagenesis and transgenic assays were used to understand whether the EGF motif of the zebrafish S-vcanb involved in Amiodarone inhibit cancer cells metastasis. The S-vcanb EGF motif contains eight cysteine residues that form specific disulfide bridges responsible for the secondary structure of the motif. As report by Schrijver *et al*. (31), eight Cystenines were mutated to Arginine using six primer pairs to disrupt the formation of disulfide bridges. These eight primers were: *s-vcanb* mE P (CAC GTGGTGCAAAAGA TGTTGCGTACCCAATGATTG), *s-vcanb* mE L1 (ACGTT GTTCACCACTGTATCCAGGTTGACGTATGCGGAT), *s-vcanb* mE L2 (GTGACGTTCATCAATGTCTATTTC ACGTTGTTCACCACT), *s-vcanb* mE L3 (ATTACGA CGAGGGTTTGTGTGACGTTCATCAATGTCTAT), *s-vcanb* mE R1 (ATTGATGAACGTCACACAAACCCTC GTCGTAATGGA GGA), *s-vcanb* mE R2(CGTCGTA ATGGAGGAACACGCATTGATGGC CTAAACTCA), *s-vcanb* mE R3 (GATGGCCTAAACTCATTCACCC GTCTA CGTCTTCCA), and *s-vcanb* mE K (GGTACCT GATCAGGGAGTTTATCT CATAGCGTGGAC).

### Wound-healing assay

Melanoma cell lines B16OVA, and breast cancer lines JC and 4T-1 were individually seeded at a density of 1 × 10^6^ onto 6-well plates in DMEM/RPMI 1640 medium and maintained at 37°C until they reached 95% confluence. The cell monolayer was wounded by a sterile pipette tip to create a 1-mm cell-free path. Culture medium was removed, and the samples were washed with PBS, followed by culturing in DMEM/RPMI 1640 medium with 10 μg/ml Mitomycin C. Cells were photographed under a low-magnification microscope. The wounded cultures were incubated in medium with or without 15 μM Amiodarone, followed by photography. The distances between the wounding center and the front of the migrating cells (vertical axis) were measured for statistical analysis.

### Matrigel invasion assay

DMSO-treated control cells and Amiodarone-treated cells were seeded in serum-free media and were trypsinized, and 0.1 × 10^6^ cells plated on transwell chambers precoated with 20 μg Matrigel. Medium-containing 10% FBS in the lower chamber served as chemo-attractant. After 12/24 h, non-invading cells were removed with cotton swabs. Invading cells were trypsinized and counted.

### Western blot analysis

Cultured cells were harvested by trypsin-EDTA, washed once with cold PBS and lysed in radioimmunoprecipitation (RIPA) buffer (Thermo scientific). Total proteins extracted from cells were analyzed on a 12% SDS-PAGE gel, and Western blot analysis was performed using antiserum against Versican V1 (Abcam; 1:1,000 dilution), Versican V2 (Thermo; 1:1,000 dilution), AKT (Cell Signaling; 1:1,000 dilution), pAKT (Cell Signaling; 1:1,000 dilution), ERK (Cell Signaling;1:1,000 dilution), pERK (Cell Signaling; 1:1,000 dilution), Snail (Cell-Signaling; 1:1,000 dilution), Twist (Santa Cruz; 1:1,000 dilution), E-cadherin (BD, 1:10,000 dilution), N-cadherin (BD; 1:1,000 dilution), GSK3β (BD; 1:1000 dilution), pGSK3β (Cell Signaling; 1:1,000 dilution), EGFR (Santa Cruz; 1:200 dilution), pEGFR (Y845) (Cell Signaling; 1:1,000 dilution), pEGFR (Y1173) (Cell Signaling; 1:1000 dilution), GAPDH (Santa Cruz; 1:1,000 dilution), and α-tubulin (Sigma; 1:1,000 dilution).

### Immunohistochemistry (IHC)

Fresh breast tumor tissue was immediately placed in formalin fixative and paraffin embedded. Four-to-six-micrometer sections were cut from the tissue blocks and mounted on charged slides. The sections were de-paraffinized with xylene and ethanol and subjected to heat-induced epitope retrieval by pressure cooker. After washing with Tris-Buffered-Saline (TBS) containing 0.025% Triton X-100, the sections were blocked with 10% goat serum and incubated with primary antibodies against VcanV1, VcanV2, Ecadherin and Twist in TBS containing 5% bovine serum albumin (BSA) overnight. The sections were washed and labeled with biotinylated secondary antibody, followed by avidin conjugated horse–radish peroxidase provided by the Vectastain ABC kit (Vector, PK-4000). The staining was developed by DAB kit (Vector, SK-4100). The slides were subsequently stained with Mayer's Hematoxylin for counter staining followed by slide mounting.

### MTT assay and amiodarone dose optimization

Cells were plated at a density of 1 × 10^4^ per well in 96-well plates and incubated for 12–16 h to allow cells to adhere. After 24 h, 0.5 mg/mL 3-(4,5-dimethyl-2-thiazolyl) -2,5-diphenyl-2H-tetrazolium bromide (MTT; Sigma) was added, and the cells were incubated for 4 h. After incubation, 100 μl of 10% SDS/0.01N HCl solution were added to each well, followed by shaking for 5 min. Then, the absorbance was measured at 570 nm. Results are representative of three independent experiments, each done in quintuplicate. Since *in vitro* studies have reported that Amiodarone may be cytotoxic, we performed an MTT assay to determine the least cytotoxic concentration of Amiodarone. Amiodarone was tested at concentrations ranging from 5 to 30 μM. 15 μM was the highest concentration at which all three cell lines tested maintained viability (Supplementary Figure S1). Therefore, 24 hr treatment with 15 μM Amiodarone was used throughout the subsequent cell based, *in vitro* studies experiments. For other drug treatments, 20 or 50 mg/ml EGF (Peprotech) or (2′*Z*, 3′*E*)-6-bromoindirubin -3′-oxime (BIO; 1 μM; EMD Biosciences) were treated with cells for 1 hr, wash out by PBS buffer several times and then for further experiments. AG-1478 (30 uM; Merck), Cetuximab (75 μg/ml; 32; Merck) and Dronedarone (5 μM; Sigma) were treated with cells for 24 hr.

### Animal studies

For experimental metastasis assays, female Balb/c mice (8–10 weeks old) were used. 2.5 × 10^5^ 4T-1 cells were suspended in 0.1 ml PBS and injected into the lateral tail vein. Animals were then treated with either placebo (PBS), tamoxifen (400 mg/kg/day) or amiodarone (180 mg/kg/day) per oral route. Lung metastatic progression was monitored by quantification of nodules at the time of euthanasia, typically at 3 weeks post inoculation. All animal experiments were in accordance with a protocol approved by the National Defense Medical Center Institutional Animal Care and Use Committee with ethics approval number: IAUCU-13-123.

### Statistical analysis

Data are expressed as mean ± SD of three independent experiments. Differences were analyzed by Student's *t*-test. *P < 0.05, **P < 0.01 and ***P < 0.005 indicated the levels of significant difference.

## SUPPLEMENTARY FIGURES


