# Supplementary material for: Cancer metastasis and EGFR signaling is suppressed by amiodarone-induced versican V2
Source: Oncotarget. 2015 Oct 21;6(40):42976–87. doi: 10.18632/oncotarget.5621 (PMC4767485; doi:10.18632/oncotarget.5621)
Supplement: Supplementary file 1 [file oncotarget-06-42976-s001.pdf]

SUPPLEMENTARY FIGURES

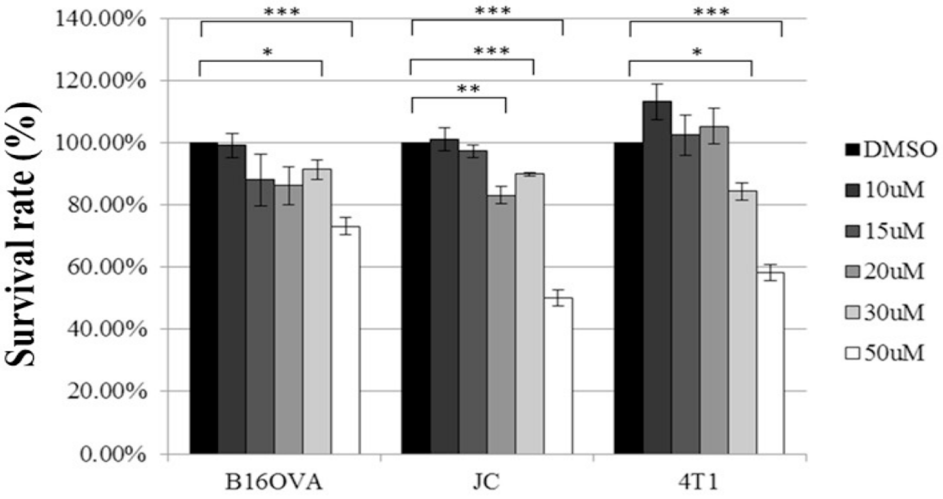

Supplementary Figure S1: Low dose of Amiodarone did not influence B16OVA, JC and 4T-1 cells survival. The cell survival assay to measure drug-induced cytotoxicity was examined by MTT following 24 hr treatment of 10 to 50 µM Amiodarone. Data is represented as a percentage of the DMSO control (DMSO), which was set to 100% and is expressed as mean ± SEM (*n* = 3).

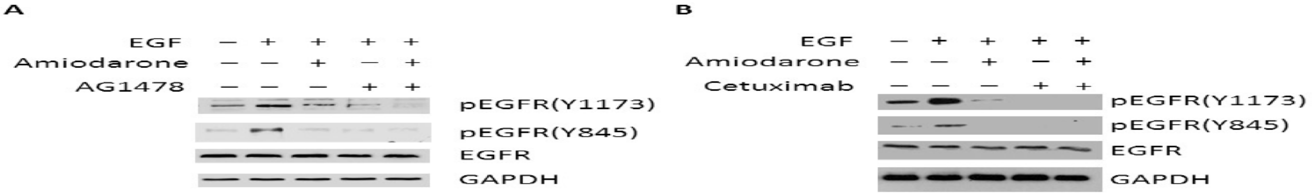

Supplementary Figure S2: Loss of function assay to prove that Amiodarone can repress EGFR activity. A. Human MDA-MB-231 cells were treated with EGF (50 ng/ml) for 1 hr and then treated with Amiodarone (15 uM), AG1478 (30 µM) or both for 24 hr. Western blot analysis of pEGFR (Y1173), pEGFR (Y845) and total formed of EGFR in these cells. **B.** MDA-MB231 cells were treated with EGF (50 ng/ml) for 1 hr and then treated with Amiodarone (15 uM), Cetuximab (75 µg /ml) or both for 24 hr. Western blot analysis of pEGFR (Y1173), pEGFR (Y845) and total formed of EGFR in these cells. GAPDH were used as internal control.

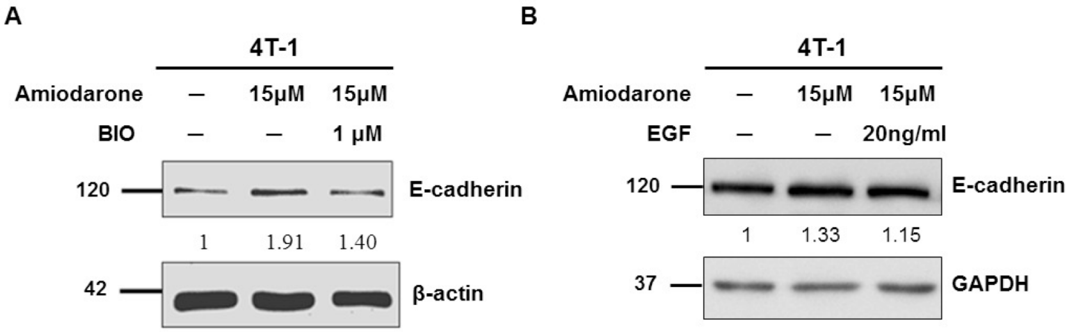

Supplementary Figure S3: Amiodarone influence EMT marker through EGFR/GSK3β pathway. 4T-1 cells were pre-treated with DMSO (-), BIO **A.** or EGF **B.** for 1 hr, and then add 15 μM of Amiodarone for 24 hr. Western blot analysis of E-cadherin in each group as indicated. The GAPDH and β-actin were used as internal control. The relative intensities of each protein were as indicated.

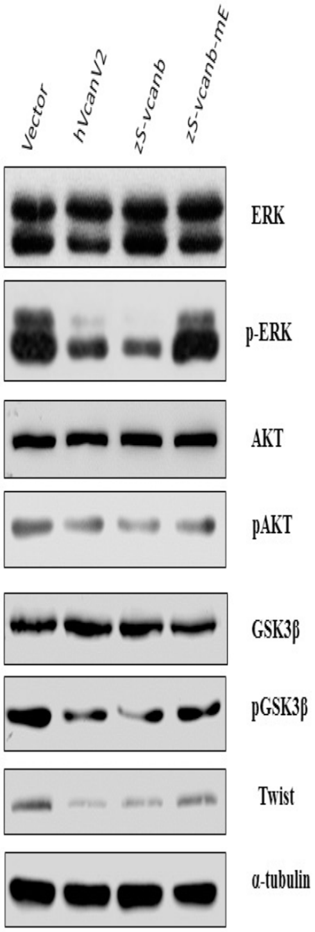

Supplementary Figure S4: VcanV2 regulate EGFR downstream signaling and EMT marker. Western blot analysis of pERK (T202/Y204), ERK, pAKT (Ser473), AKT, pGSK3β (Ser9), GSK3β and twist in human Versican V2 (hVcanV2), zebrafish S-vcanb (zS-vcanb), and zebrafish S-vcanb-mE overexpressed cells.

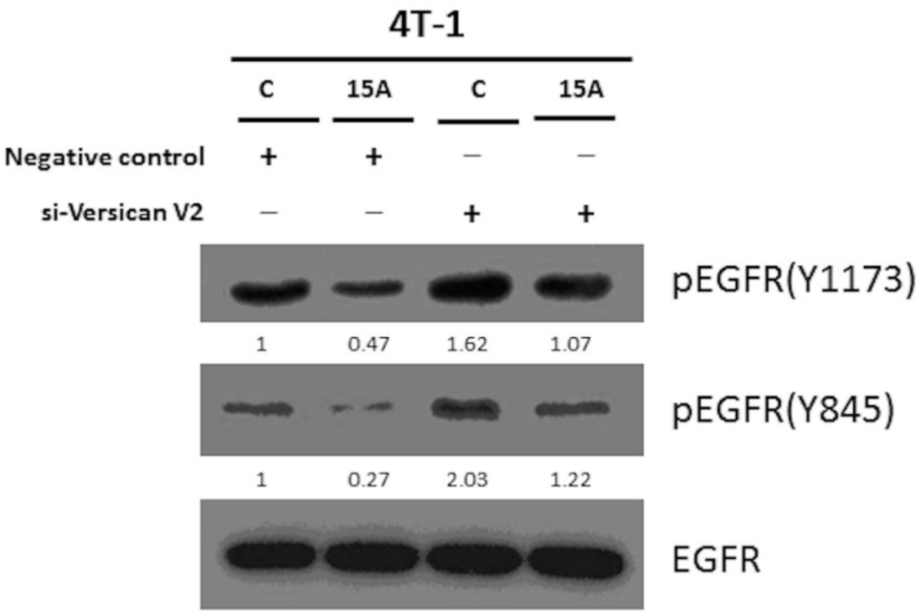

Supplementary Figure S5: The effects of Amiodarone on inhibiting EGFR activity was dependent on VcanV2. Western blot analysis of pEGFR (Tyr845), pEGFR (Tyr1173) in control cells and VcanV2 knockdown cells treated with DMSO (c) or 15 μM Amiodarone (15A).

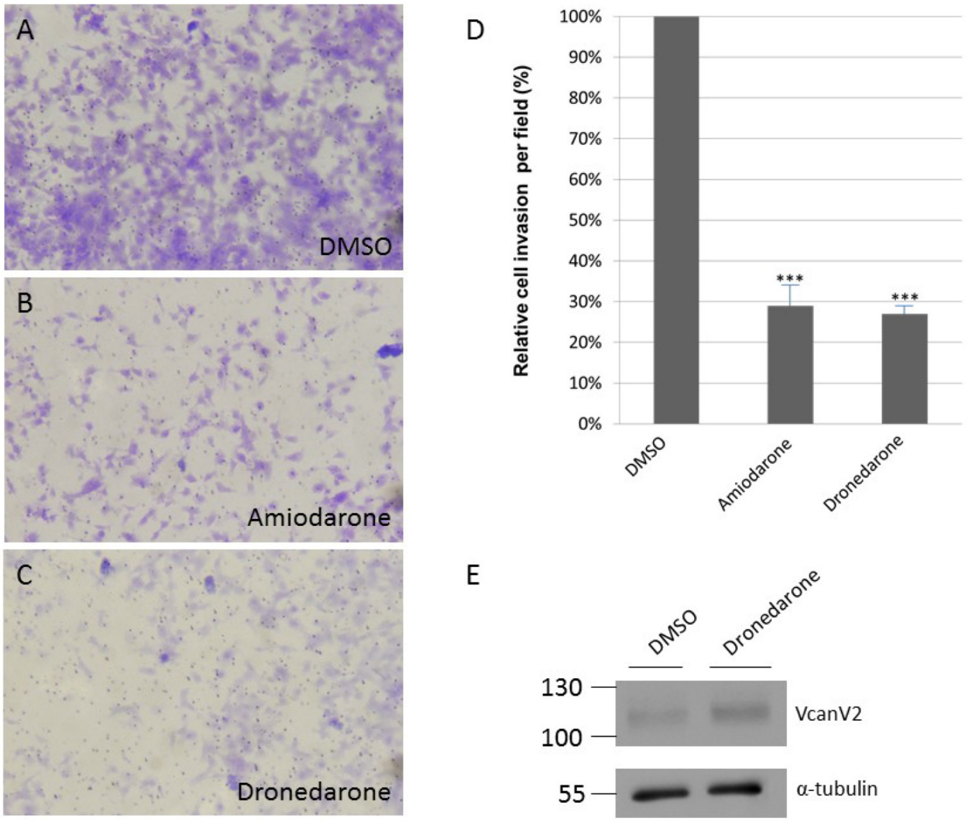

Supplementary Figure S6: Dronedarone repress tumor cell metasis and induced VcanV2 expression. Matrigel invasion assay of 4T-1 cells treated separately with **A.** DMSO, **B.** 15 µM of Amiodarone and **C.** 5 µM of Dronedarone. **D.** Quantification of matrigel invasion. Data were representative as mean ± SEM which was the average of three independent experiments. Each was obtained from counting five randomly selected fields. Statistical significance was determined by Student's *t*-test. ***$P < 0.001$. **E.** Western blot analysis of VcanV2 in control (DMSO) or Dronedarone (5 µM) treated cells. A-tubulin was used as internal control.
